# Feasibility Study of a Wearable System Based on a Wireless Body Area Network for Gait Assessment in Parkinson's Disease Patients

**DOI:** 10.3390/s140304618

**Published:** 2014-03-07

**Authors:** Jorge Cancela, Matteo Pastorino, Maria T. Arredondo, Nikita S. Konstantina, Federico Villagra, Maria A. Pastor

**Affiliations:** 1 Campus de Excelencia Internacional (CEI) Moncloa, Universidad Politecnica de Madrid (UPM)-Universidad Complutense de Madrid (UCM), Ciudad Universitaria, Madrid 28003, Spain; 2 Life Supporting Technologies Group, Universidad Politecnica de Madrid (UPM), Ciudad Universitaria, Madrid 28003, Spain; E-Mails: mpastorino@lst.tfo.upm.es (M.P.); mta@lst.tfo.upm.es (M.T.A.); 3 Biomedical Simulations and Imaging Laboratory, School of Electrical and Computer Engineering, National Technical University of Athens, Iroon Polytechniou 9, Athens 15780, Greece; E-Mail: knikita@ece.ntua.gr; 4 Division of Neurosciences, Center for Applied Medical Research (CIMA), University of Navarra, Pamplona 31008, Spain; E-Mails: fvillagra@unav.es (F.V.); mapastor@unav.es (M.A.P.)

**Keywords:** gait assessment, Parkinson's disease, wearable body area network, accelerometers

## Abstract

Parkinson's disease (PD) alters the motor performance of affected individuals. The dopaminergic denervation of the striatum, due to substantia nigra neuronal loss, compromises the speed, the automatism and smoothness of movements of PD patients. The development of a reliable tool for long-term monitoring of PD symptoms would allow the accurate assessment of the clinical status during the different PD stages and the evaluation of motor complications. Furthermore, it would be very useful both for routine clinical care as well as for testing novel therapies. Within this context we have validated the feasibility of using a Body Network Area (BAN) of wireless accelerometers to perform continuous at home gait monitoring of PD patients. The analysis addresses the assessment of the system performance working in real environments.

## 1. Introduction

Parkinson's disease (PD) is the second most common neurodegenerative disorder after Alzheimer's disease and it is expected to impose an increasing social and economic burden on society in the coming decades. The prevalence of PD in industrialized countries is generally estimated at 0.3% of the entire population and about 1% in people over 60 years of age. Reported standardized incidence rates of PD are 8–18 per 100,000 person-years. Onset of PD is rare before age 50 and a sharp increase of the incidence is seen after age 60 [1]. About 20% of people over the age of 80 have Parkinsonism-associated gait disturbances. The major motor disturbances in PD are bradykinesia (*i.e.*, slowness of movement), hypokinesia (decreased movement amplitude), resting tremors, rigidity, and postural instability [2]. These major motor features of PD are associated with, and are largely a result of, the loss of dopaminergic innervation of the basal ganglia. Although a genetic predisposition has been identified in a subset of patients with PD, several other risk factors for PD have been recognized [3–5]. The cause and etiology of PD are still unknown [3–7]. In addition to multiple other effects, the impaired basal ganglia function in PD leads to alterations in gait and balance. These motor changes in PD often restrict functional independence and are a major cause of morbidity and mortality among these patients [8–11]. PD is typically characterized by severe, unpredictable and abrupt changes in the patient motor performance whereby OFF periods, characterized by a drug's effectiveness wearing off, alternate with ON periods, during which medication effectively improves movement.

These motion changes can be detected by studying the variation of the signals recorded by accelerometers attached in the limbs and belt of the patients. Furthermore, the analysis of the most significant changes in these signals makes possible to build an individualized profile of the disease, personalize the medication intakes and improve the response of the patient to the treatment [12].

Within this work we analyze the feasibility of using a Body Network Area (BAN) of wireless accelerometers to perform a continuous gait monitoring of PD patients at their homes. The development of a reliable tool for the continuous monitoring of gait in PD patients would provide several benefits for PD patients and for the general practice: (1) in the long-term, a better understanding of the disease evolution on every patient and an indicative of the PD progression; and (2) in the short-term, the detection of abrupt changes in the gait measures on a daily basis could lead to the identification of wearing off phases. In both cases, the identification of such events (PD progression and/or wearing off phases) will contribute to enrich the clinical decision making process with more and more reliable data.

## 1.1. Gait Disturbances in Parkinson's Disease: Classification

The gait disturbances in PD may be divided into two types [3]: (1) continuous, mainly characterised by a reduction of gait speed [13] and (2) episodic [14,15]. The episodic gait disturbances occur occasionally and intermittently and appear randomly. The episodic gait disturbances include festination, gait initiation hesitation, and freezing of gait [9,16–18]. Freezing of gait is an incapacitating phenomenon that is experienced mainly by patients with advanced PD [9,19–21]. The continuous changes refer to alterations in the walking pattern (temporal and spatial kinematic parameters). Both types of disturbances are due to dysfunction of the basal ganglia, although the mechanisms for such disturbances are independent and they are responsible for the increase in the incidence of falls in PD patients [9]. Falls are one of the most significant consequences of a disturbed gait in PD [9,17,22,23]. As the disease progresses, gait impairment and falls become increasingly important and develop into one of the main complaints among PD patients and caregivers. The most relevant changes (temporal and spatial) affected by PD are apparent only when gait is evaluated quantitatively with gait analysis systems. Increased left-right gait asymmetry and diminished left-right bilateral coordination are changes affected by the disease [22,23]. Another gait feature in PD patients seems to be the inability to generate a consistent and steady gait rhythm, resulting in an increase in higher stride-to-stride variability [24–26]. An increase of gait variability can be detected throughout the disease even in early the stages of the disease when patients have not started taking anti-Parkinsonian medications [25]. The magnitude of the variability is enhanced by disease severity. It has been shown the relationship between gait variability, fall history and other Parkinsonian features [26–29]. An effect of levodopa administration has been described on gait variability and fall frequency in PD patients [30]. In the OFF state, stride time variability was significantly larger among fallers compared to non-fallers [28]. Stride time variability decreased significantly in response to levodopa in both groups (fallers and non-fallers) [30]. However, in the ON state, stride time variability remained significantly higher in the fallers than non-fallers. The locomotor control system that regulates gait variability and gait phases timing is impaired in PD patients with a history of falls [28]. On the other hand, no significant correlation has been described between stride-to-stride variability with other motor features such as tremor, rigidity, or bradykinesia in the OFF state [28]. In addition, levodopa decreases stride-to-stride variability in non-fallers, suggesting that dopaminergic networks regulate the control of gait variability and timing suggesting the possibility of damaged and exaggerated impairment of “internal clock” function in PD fallers [28]. In the ON state, when the motor performance is optimal, the PD fallers showed also a further increased control of stride-to-stride variability. The authors of [28] suggest the possibility of damaged and exaggerated impairment of “internal clock” function in PD fallers. In addition Parkinson's disease patients have shown impaired visual sampling during gait through complex environments. They had fewer early preparatory saccades recorded than controls preceding turns and under dual-task conditions made less frequent saccades than controls [31].

## 1.2. Parkinson's Disease Monitoring

The evolution of wearable sensors and systems during the last decade, introducing new capabilities and extending the functions of existing ones, has led to the development of a wide range of tools and services for the patient home monitoring. Neurodegenerative disorders, such as Parkinson's Disease, have also benefited from these advances [32]. The development of a reliable quantitative tool suitable for continuous monitoring able to evaluate the motor performance evolution, as well as sudden changes from ON-OFF state, would be an important step forward both for routine clinical care as well as for trials of novel therapies, *i.e.*, drugs or devices. Gait performance deterioration is one of the major symptoms of PD and it is composed of different elements, *i.e.* freezing of gait, gait, bradykinesia and postural instability [2]. Due to such complexity, gait disorders reflect important pathological mechanisms underlying PD and therefore they are a good model for a quantitative estimation. Several works have addressed these issues using wearable and wireless technologies. Tien *et al.* [33] have developed a wireless inertial sensor system to characterize gait abnormalities in PD by analyzing physical features such as pitch, roll, and yaw rotations of the foot during walking. Then, the Principal Component Analysis (PCA) technique was used to select the best features, and finally a classification model was built using a Support Vector Machine (SVM). Results have demonstrated the ability of successfully detect the presence of PD based on physical features of gait. In [34] researchers have used a miniaturized triaxial accelerometer-based system for the detection of gait and postures concluding that a triaxial monitor system is a practical and valuable tool for objective, continuous evaluation of walking and postures in patients with mild to moderate PD. Wearable sensors have also been integrated with web-based applications [35–38] enabling home monitoring of patients with (PD) using wearable sensors. This web application offers three different options: a resource-aware data collection engine that relies upon wearable sensors, web services for live streaming and storage of sensor data, and a web-based graphical user interface client with video conferencing capability. Reference [39] has suggested a system for the early automatic recognition of health problems that manifest themselves in a distinctive form of gait. The purpose of the system is to prolong the autonomous living of the elderly at home. The gait of the elderly user is captured using a motion-capture system, which consists of body-worn markers and wall-mounted sensors. A triaxial accelerometer was also used in the lower back to measure the variability (consistency and rhythmicity) of stepping [40] or to evaluate parameters derived from accelerometry data of gait in different neurological conditions with pathological gait impairment compared to healthy subjects [30]. Finally, in the work described in [41] subjects have performed standardized gait tests while wearing sport shoes equipped with inertial sensors (gyroscopes and accelerometers); signals were recorded wirelessly, features were extracted, and distinct subpopulations were classified, showing that it is possible to distinguish mild from severe gait impairment.

## 2. Experimental Section

## 2.1. Framework

**Figure 1.**
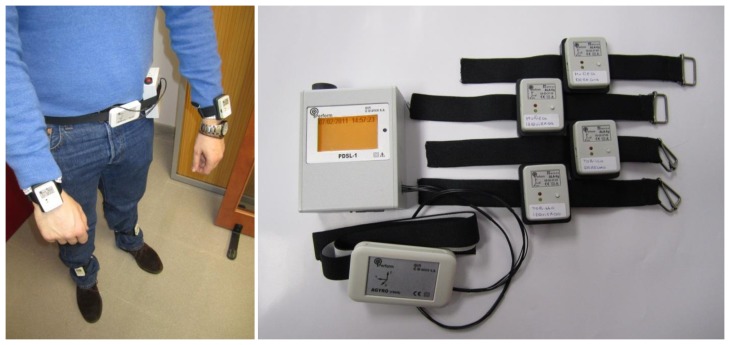
Sensors and data logger used for data collection and their position on the body.

“A sophisticated multi-parametric system FOR the continuous effective assessment and Monitoring of motor status in Parkinson's disease and other neurodegenerative diseases” (PERFORM) is a telematic platform for remote PD monitoring developed during the last years by a European Consortium of Small and Medium Enterprises (SMEs), large companies, Universities and research centers [12,42]. The current status of the project is a fully operative prototype which has been tested in three different hospitals across Europe: University of Navarra Medical School Hospital (Spain), the University of Ioannina Hospital (Greece) and the Nuovo Ospedale Civile S.Agostino-Estense of Modena (Italy). The PERFORM platform is composed by a set of four tri-axial accelerometers used to record the (3-axis) ccelerations of the movements at each patient limb and one accelerometer and gyroscope used to a record body accelerations and angular rate attached on the belt of the patient. Sensors were placed in every limb and belt to detect and quantify a wide range of symptoms related to Parkinson's disease, *i.e.*, tremor, bradykinesia, dyskinesias, falls and freezing of gait (Figure 1).

The four sensors in the limbs transmit data using the Zigbee protocol to a data logger device located on the belt of the patient, which receives and stores locally all the received signals. The sampling rate used was 62.5 Hz (16 milliseconds between each sample). This set of accelerometers and data logger were designed, developed and manufactured according to the requirements of the PERFORM project. In that sense, the sampling rate was fixed in 62.5 Hz due to the available technology, literature review and preliminary tests carried out within the first years of the project where such sensors were validated. It is important to highlight that the sensors do not need to be placed in a precise position since the system will work with the module of the 3-axis acceleration signal, not with a particular axis. The output of each axis is the acceleration in such direction and it ranges from −6 g to +6 g. It is possible to wear the devices without any help, nevertheless, some PD patients could require a caregiver's help (depending on the patient mobility). The only requirement is to adjust the sensors as tightly as possible. These accelerometers do not provide any retransmission protocol; but all the transmitted samples incorporate a unique timestamp. Using this this timestamp it is possible to evaluate in the receptor if any packets was lost during the transmission. Apart from the wearable devices, each patient was provided with touch-screen PC at his/her home. The application installed in the PC carries out the signal processing tasks. At the end of the day, the logger is connected to the PC through a standard USB connection and all the data is automatically transferred to the computer. Once the raw data is downloaded from logger to the PC, it is automatically processed. This software is responsible for the identification and quantification of the patient. A customized graphical user interface (GUI) has been designed to allow the patients enter other useful information. The user could interact with it either with the mouse or with the touch screen modality. Using a touch-screen system offers interesting benefits in the PERFORM case [43]. Touching a visual display of choices requires little thinking and it is a form of direct manipulation easier to learn. Touch screens have more intuitive hand-eye coordination than mice or keyboards. Consequently, touch screens are the fastest pointing device. Besides, the GUI was tested on every phase of the pilots and redesigned according to the users' feedback. The software allows the patients to insert the following information:

- medication intake (type, dose and time)
- meals (type of food, amount, time)
- PDQ-39. An standard questionnaire for the evaluation of physical, emotional and psychosocial aspects of Quality of Life (QoL) in PD patients [44].

By definition, working on unsupervised environments implies an important number of challenging problems regarding the signals interpretation and signals quality validation [45]. Collecting this information is essential in order to create a context and to make the signal processing outcome useful in this sort of environments. In fact, motor behavior strongly depends on the assumption of the medication (in the usual patient's dosage) and the metabolism of the drug is influenced by the diet (proteins or fats).

## 2.2. Participants

Patients fulfilling the following criteria were eligible for the study: a diagnosis of Parkinson's disease, aged between 40 and 70 years old, ambulatory, capable of complying with study requirements, receiving stable dopaminergic treatment, experiencing motor fluctuations and being supported by a responsible caregiver who can cooperate with patient and doctor. Participants suffering from dementia, hallucinations or any significant systemic disease were excluded from the study. Table 1 shows the assessment of bradykinesia, gait and limbs' rigidity according to the Unified Parkinson's Disease Rating Scale (UPDRS) [46], this evaluation was done by a clinician on the first day of the test.

Before taking part to the study, patients were provided with a participant information sheet describing the study in their own language as well as an oral explanation of the research expressed in terms that would have the best chance of being understood. The experimental nature of this study, its inherent risks and drawbacks, and its chance of improving the treatment of PD were discussed. Then clinician obtained informed consent and gave a letter with a synopsis of Perform Protocol for family doctor to inform him about main issues of the study.

## 2.3. Data Collection

**Figure 2.**
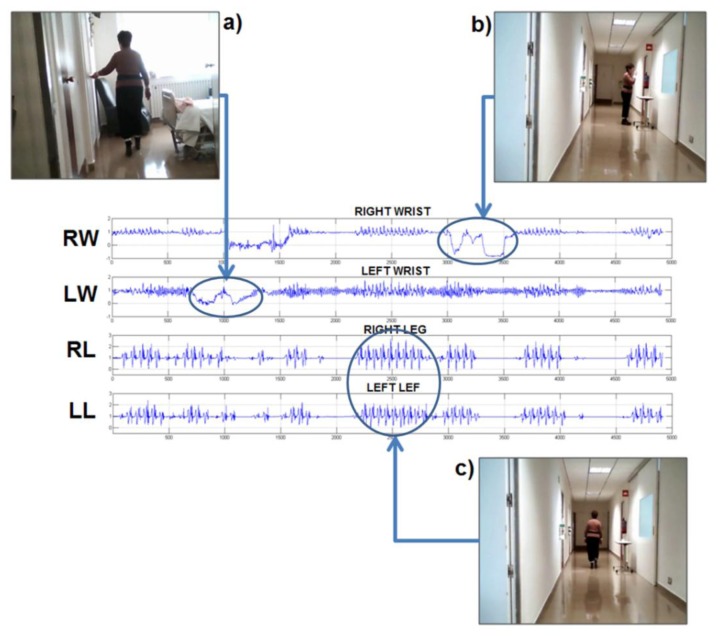
This figure shows raw data coming from the four accelerometers in the limbs. The module of the 3-axis sensors is plotted to show how each of these signals change when the subject perform different daily tasks. Signals RW (right wrist) and LW (left wrist) show the acceleration in the wrists and RL (right leg) and LL (left leg) acceleration in the legs. Panel (**a**) shows the subject opening a door with her left hand and the arrow links this moment with the raw data signal. Panel (**b**) shows the subject moving her right hand to drink water and the corresponding raw signal. Panel (**c**) shows a moment where the patient was walking. The arrows relate the tasks with the response in the signals. The PERFORM system has its own activity recognizer module which is based on analysis of these signals as explained in [48].

The PERFORM system was installed at patients' homes. All patients were asked to wear the system in their homes and to move freely carrying out their daily activities. The patients were using the system between 5 and 7 days, running two sessions of 4 h each per day, Figure 2 shows an example of raw data collected during these sessions. During the first day, they and their caregivers received a training session about how to use properly the system and the sensors. Moreover, a printed manual was left at their homes and a telephone line was available for questions during the whole time as well. On the last day, both the patients and caregivers were interviewed to report problems or issues related with the use of the system. The guidelines to for the interview were extracted from the work of Knight *et al.* [47], who suggested a methodology to assess the comfort of wearable computers.

## 2.4. Data Analysis

People suffering from PD are unable to move fluently and that modifies the walking pattern generating a more complex and entropic signal. This approach constitutes an important tool for the detection of a PD walking pattern. We can see that *x*-axis (corresponding with the cranio-caudal axis) and *z*-axis (corresponding with the anterio-posterior axis) contain most of the information related with the walking movement. During forward movement and as consequence of each step the, acceleration along the *z*-axis increases and decreases following the gait cycle.

Two different types of analysis are performed; the first one using intrinsic features of the signal, specifically entropy, which by definition is an excellent feature to measure complexity. Entropy is the measure of the uncertainty or unpredictability associated to a specific variable, or in other words, it is a measure of the disorder. Former works [49–51] have shown how to use the technique “Sample Entropy” to calculate the variability and complexity of gait in PD disease. Sample entropy quantifies the regularity of a time series. It reflects the conditional probability that two series of “m” consecutive data points which are similar to each other will remain similar when one more consecutive point is included [52]. Two data series are considered similar if the value of a specific measure of distance is less than a parameter “r”.

**Figure 3.**
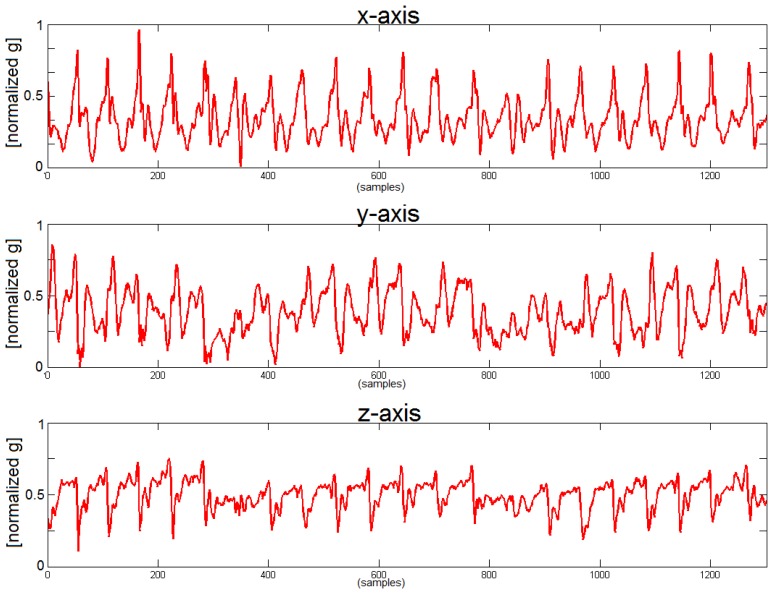
Signals from the belt sensor in a healthy subject. From top to bottom the figure shows the *x*-axis, *y*-axis and *z*-axis. Values on the horizontal axis are samples and vertical axis is the normalized output of the accelerometers [49], the acceleration value of the sensors range from −6 g to +6 g, the vertical axis of the figure shows the values normalized between 0 and 1 by subtracting the minimum (−6 g) and dividing by the range (12 g).

**Figure 4.**
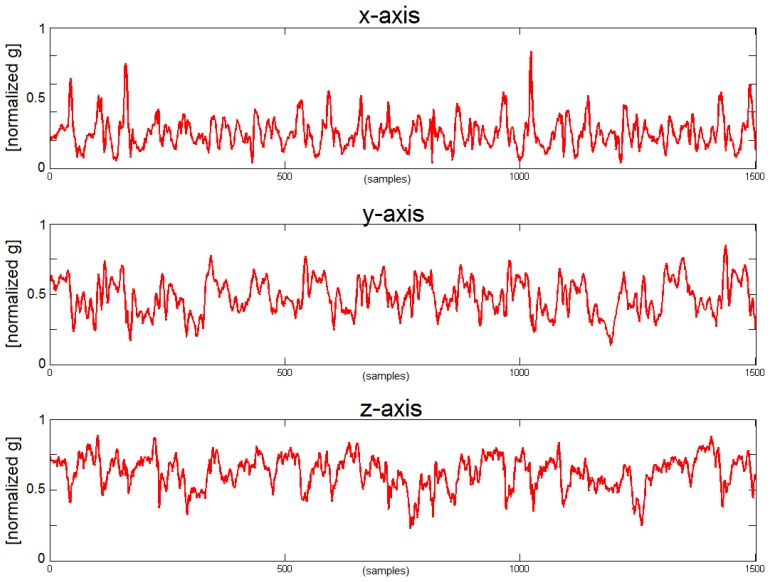
Signals from the belt sensor in a PD patient. From top to bottom the figure shows the *x*-axis, *y*-axis and *z*-axis. Values on the horizontal axis are samples and vertical axis is directly is the normalized output of the accelerometers [49], the acceleration value of the sensors range from −6 g to +6 g, the vertical axis of the figure shows the values normalized between 0 and 1 by subtracting the minimum (−6 g) and dividing by the range (12 g).

On the other hand, “secondary measures” or classic gait parameters *i.e.*, step frequency, velocity, stride length are also evaluated. To estimate the stride length we use the “inverted pendulum model of human walking” [53], with an average error in the step frequency characterization of 1.88% [49]. The limitation of these features in an unsupervised environment is that they can change not necessarily because of a worsening on the patient status but for the willingness of the patient, *i.e.*, the patient at home can walk slowly or run depending on the situation. For this reason we include extra measures able to estimate the worsening of the walking pattern independently of the patient activities, the measure of the entropy of the accelerometer signal has been proved to be one the best indicators to discriminate between a “healthy” pattern and a walking pattern coming from a PD patient [49]. Actually, this result is completely aligned with the discussion about the Figures 3 and 4.

In addition, the analysis of the movement pattern in healthy subjects helps to establish a comparison with the output of the PD's patient recording. Figures 3 and 4 show the acceleration in the belt sensor for each axis in a healthy subject and in a PD patient and it is possible to see the degradation of the gait pattern in a PD patient. Finally, it is necessary to point out that a segmentation module was developed within PERFORM in order to analyze when the patient was actually walking [48] and run the gait characterization algorithms only at that times. This work has proved that even two accelerometers are enough to classify walking activities with higher than 99% accuracy.

## 3. Results and Discussion

Working in an unsupervised and wireless environment achieving a low data loss rate became essential. In the last release of the PERFORM platform, the four accelerometers in the limbs work simultaneously and transmit data in separate time slots avoiding most of the packet loss. Nevertheless sometimes there could be something between the sensor and the receiver, *i.e.*, when patient is walking the body is between the sensor and the receiver and that could generate some packet loss.

Table 2 shows the average data loss and standard deviation, the average length of data loss burst and standard deviation and the most frequent length of data loss burst. The average data loss satisfied that in all cases more than 95% of the data is successfully transmitted. Nevertheless, analyzing the average burst length and the standard deviation of these values we found burst of up to 143.04 ± 240.10 ms, a burst of these characteristics in the middle of the transmission would compromise seriously the performance and accuracy of the system. Nevertheless, adding the most frequent burst length as an extra measure and observing the raw data we can easily identify that 64 ms is the most common burst in all the cases, and moreover, the longest bursts are always at the beginning of the session, *i.e.*, during the startup process. That means that waiting for all the sensors to be properly connected before start computing the gait features will remove the longest burst errors. Then, we will still keep sporadic disconnections of 64 ms (the minimum time for the data logger to reconnect with the accelerometer) which will still guarantee a much more acceptable average data loss.

The interview with patients and caregivers after using the system for a week also resulted in interesting comments. The majority of the patients, two thirds, did not feel any discomfort/pain at most of their body parts. Only, one third of the patients felt extremely week discomfort/pain at their body. None of the patients had the perception of any kind of harm (e.g., headache, pain, itching, irritation, *etc.*) caused by the devices. The interview revealed some emotional and appearance issues of a group of patients (27.2%). These patients had some concerns about the impression they make to others when wearing the device. They would feel much more comfortable if the device was not visible or if they had to wear it only at home. All participants agreed that the provided solution did not obstruct them in everyday activities neither limit their activities in an effective way. The only concern expressed by a significant number of patients was regarding the proper attachment of the current appliance.

## 4. Conclusions and Outlook

Home Health Monitoring is more than using wearable sensors at patients' home, especially, talking about chronic disease management. It means to involve patients and caregivers in the healthcare path and shift their role from a passive position to an active position. And therefore, transform them in main actors of the healthcare process. The first and most obvious reason is that home telehealth systems need the participation and cooperation of patients and caregivers to work efficiently. In the PERFORM context the algorithms developed have achieved a good accuracy. Nevertheless, in order to achieve a good understanding of the disease in the patient and to build a profile of the patient, context information (*i.e.*, food and medication intakes) is required. That means it is necessary to invest time and effort in the patient and caregiver training, to provide them with the adequate skills to use the system properly, as well as follow the best practices for GUI design. Moreover, it is important to make them aware of the importance of the self-assessment, not only for a reliable remote monitoring but also for improvement of their disease management. It is important to keep in mind that different subjects perform different walking patterns and even the same subject will walk differently depending on the situation. The lack of contextual information makes working in unsupervised environments a very challenging task. Parameters related to the walking analysis are not fully representative (speed and stride length are features which need to be in a context, we need to be aware of the fact that sometimes the patient could walk slower than usual because he wants to, e.g., because he is relaxed at home) and the use of alternative measures like entropy are a better choice. As a general recommendation the involvement of an active patient becomes crucial in order to contextualize as much as possible the raw biomedical signals. From this experience, it is also important to remark the importance of working in more accurate activity recognizers, as well as affective recognizers able to segment the activities and moods of the subject during their daily life.

Studying the walking pattern on PD patients is a promising tool in order to develop a continuous monitoring system able to identify the different phases of the disease within the day (OFF-ON). This system could easily alert the professionals when the patient faces an OFF phase, measuring the degradation of the walking pattern, indicating that a schedule change in the medication treatment is needed.

Contextual data is crucial in order to be able to interpret and contextualize the patient gait performance. The use of a patient diary provides excellent information to correlate with the movement assessment, and the new generation of smartphones is a convenient platform to integrate the patient diary and the data collection coming from the limb sensors. Likely the future of this technology will move to the use of these platforms.

## Figures and Tables

**Table 1.** Details of the patients enrolled in the feasibility study. Table shows the sex, age and clinical evaluation of the patients during their ON and OFF states according to the UPDRS scale [46].

**Id**	**Sex (Age)**	**Status**	**Gait**	**Bradykinesia**	**Left leg rigidity**	**Right leg rigidity**	**Left wrist rigidity**	**Right wrist rigidity**
1	F (71)	OFF	0	1	1	1	1	1
		ON	0	1	1	0	1	0
2	M (68)	OFF	0	0-1	1–2	0–1	1	0–1
		ON	0	0	0	0	0	0
3	M (63)	OFF	0	0	1	1	1	1
		ON	0	0	0	0–1	0	1
4	F (67)	OFF	3	2	2	2	1	2
		ON	1	1	1	1	1	1
5	M (63)	OFF	0	0	0	1	0	2
		ON	0	0	0	1	0	2
6	M (68)	OFF	0	3	0	0	1	1
		ON	0	1	1	2	1	1
7	M (76)	OFF	0	1	1	0	0	1–2
		ON	0	0	1	0	1	2
8	M (52)	OFF	4	3	4	4	4	4
		ON	1	1	1	1	1	1
9	F (56)	OFF	2	3	3	2	3	2
		ON	1	0	1	0	1	0
10	M (58)	OFF	2	3	3	3	3	3
		ON	1	1	1	1	1	1
11	F (79)	OFF	0	2	1	1	1	1
		ON	0	0	0	0	0	0

**Table 2.** Data loss measures for the accelerometer network working on a real environment.

**Id**	**Data loss (%)**	**Average burst error length (ms)**	**Most frequent burst error length (ms)**
1	0.95 ± 0.77%	111.55 ± 532.89	64
2	0.97 ± 0.55%	92.07 ± 242.97	64
3	2.02 ± 0.78%	104.22 ± 531.12	64
4	1.14 ± 1.12%	101.25 ± 532.89	64
5	1.51 ± 1.17%	114.85 ± 529.04	64
6	0.32 ± 0.37%	143.04 ± 240.10	64
7	1.83 ± 1.04%	139.44 ± 542.15	64
8	1.25 ± 1.26%	123.64 ± 242.97	64
9	1.31 ± 1.02%	174.26 ± 527.90	64
10	1.27 ± 1.07%	115.10 ± 542.15	64
11	1.84 ± 1.11%	106.55 ± 542.59	64
